# Super-resolution 2-photon microscopy reveals that the morphology of each dendritic spine correlates with diffusive but not synaptic properties

**DOI:** 10.3389/fnana.2014.00029

**Published:** 2014-05-07

**Authors:** Kevin Takasaki, Bernardo L. Sabatini

**Affiliations:** Department of Neurobiology, Harvard Medical School, Howard Hughes Medical InstituteBoston, MA, USA

**Keywords:** dendritic spine, 2-photon microscopy, stimulated emission microscopy, super resolution microscopy, synaptic transmission

## Abstract

The structure of dendritic spines suggests a specialized function in compartmentalizing synaptic signals near active synapses. Indeed, theoretical and experimental analyses indicate that the diffusive resistance of the spine neck is sufficient to effectively compartmentalize some signaling molecules in a spine for the duration of their activated lifetime. Here we describe the application of 2-photon microscopy combined with stimulated emission depletion (STED-2P) to the biophysical study of the relationship between synaptic signals and spine morphology, demonstrating the utility of combining STED-2P with modern optical and electrophysiological techniques. Morphological determinants of fluorescence recovery time were identified and evaluated within the context of a simple compartmental model describing diffusive transfer between spine and dendrite. Correlations between the neck geometry and the amplitude of synaptic potentials and calcium transients evoked by 2-photon glutamate uncaging were also investigated.

## Introduction

Dendritic spines are small, subcellular structures that house the post-synaptic machinery associated with glutamatergic synapses, typically consisting of a bulbous head joined to the parent dendrite by a channel-like neck (Harris and Stevens, 1989; Alvarez and Sabatini, 2007). The special morphology of dendritic spines suggests that they biochemically compartmentalize diffusible signaling cascades by restricting the transfer of molecules between the spine head and dendrite. Indeed, the rapid kinetics of calcium (Ca) signaling in the spine head compared to the slowed mixing of Ca across the neck under physiological conditions ensures independent signaling in each compartment for this second messenger (Sabatini et al., 2002). Such compartmentalization presumably permits the highly spatially specific induction of Ca dependent plasticity at one synapse without affecting its neighbors (Yuste and Denk, 1995; Matsuzaki et al., 2004; Harvey and Svoboda, 2007). Nevertheless, not all intracellular signals are functionally compartmentalized, and during plasticity induction, certain signaling molecules activated in the spine head are able to escape and influence neighboring synapses (Harvey and Svoboda, 2007; Harvey et al., 2008; Murakoshi et al., 2011). Thus, the ability of a biochemical signal to spread beyond the spine depends on the molecular properties of the messengers involved, mechanisms of retention and inactivation, and passive constraints imposed by the geometry of the spine and dendrite (Yasuda and Murakoshi, 2011).

In addition to biochemical signaling cascades, the excitatory synapses of dendritic spines support electrical signaling via post-synaptic potentials. It has long been hypothesized that the morphology of dendritic spines might play a role in regulating the spread of electrical signals. The opening of ion channels located in the spine, local capacitive charging of the spine head, and the electrical resistance of the neck could potentially shape the amplitude, time course, and spatial spread of synaptic potentials. However, in contrast to clear confirmation of the compartmentalization of certain biochemical signals, whether spine morphology regulates synaptically evoked electrical activity has been controversial (Tsay and Yuste, 2004). Numerous studies have examined whether the geometry of spines has, or in theory could have, a measurable impact on electrical signaling by affecting the generation and propagation of membrane potentials during synaptic transmission; the results of these studies have provided evidence supporting (Bloodgood and Sabatini, 2005; Araya et al., 2006; Bloodgood et al., 2009; Harnett et al., 2012) and rejecting (Harris and Stevens, 1989; Koch and Zador, 1993; Svoboda et al., 1996; Palmer and Stuart, 2009) a role of spine geometry in regulating the electrical function of synapses. Nevertheless, functional studies that are independent of accurate measurements of spine dimensions reveal significant drops in potential across the spine neck that alter voltage-dependent processes, such as the activation of voltage-gated ion channels and relief of the Mg block of NMDA-type glutamate receptors (Bloodgood et al., 2009; Harnett et al., 2012). Furthermore, computational models support additional potential functions of the spine morphology in normalizing synaptic potentials [for example rendering their amplitude insensitive to the morphology of the parent dendrite (Gulledge et al., 2012)] and correlative structure-function studies have found that the amplitude of synaptic potentials falls for longer spines (Araya et al., 2006).

A challenge in establishing an understanding of the structure-function relationship of dendritic spines has been an inability to obtain high-resolution structural information in an experimental context that permits functional analysis. The diffraction limited resolution of 2-photon laser scanning microscopy (2PLSM), the standard method of imaging dendritic spines in living tissue, is too coarse (~400 nm) to accurately measure the dimensions of the spine neck, which can be less than 100 nm in diameter, or fine features of the head, which can have non-spherical morphology.

We and others have implemented in-tissue superresolution 2PLSM realized via stimulated emission depletion (STED) (Ding et al., 2009; Bethge et al., 2013; Takasaki et al., 2013). STED-2P permits fluorescence imaging of neurons in brain tissue to a resolution of ~50 nm, sufficient for accurate reconstruction of spine morphology. We use this approach, coupled with electrophysiological analysis, fluorescence recovery after photo-bleaching (FRAP), and two-photon laser glutamate uncaging, to determine what features of synaptic signaling are predicted by the morphology of the spine. To preserve the highest imaging resolution, analysis was limited to spines near the surface of acute brain slices. This analysis demonstrates a clear structure-function relationship for diffusional transfer of a small molecule, such that the physical dimensions of each spine, with rare exceptions, predict the time course of diffusional equilibration across the neck. On the other hand, we find no correlation between morphology and the sizes of uncaging evoked synaptic potentials (uEPSPs) and associated Ca transients (ΔCa_uEPSP_). This latter result, given the previously established impact of spine neck resistance on synaptic signaling, suggests the possibility of counter-balanced regulation of synaptically-activated ion channels, such as glutamate receptors and voltage-gated ion channels, that normalize synaptic signals in the face of variable spine morphology.

## Materials and methods

**Figure 1:**
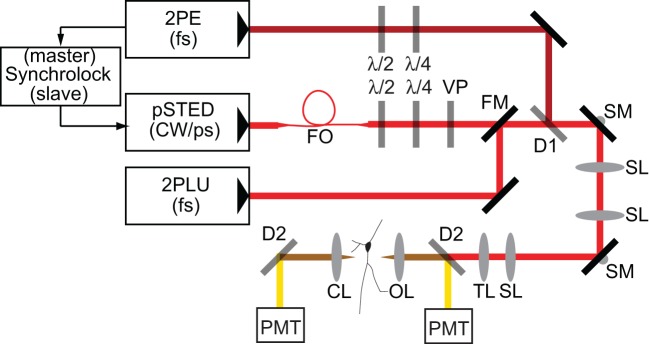
**Schematic of microscope**. Laser pulses from a femtosecond-pulsed Ti-Sapph laser tuned to 810 nm for two-photon excitation (2PE) are synchronized by an electronic feedback circuit (Synchrolock) with those of a picosecond-pulsed Ti-Sapph laser (pSTED) tuned to 736 nm for stimulated emission. The STED laser can be exchanged by a flip mirror (FM) with the beam from a femtosecond-pulsed Ti-Sapph laser tuned to 720 nm for two-photon laser-induced uncaging (2PLU) of caged compounds, such as caged glutamate. STED pulses are stretched to ~200 ps by dispersion through a 120 m single-mode polarization-maintaining fiber optic (FO) and phase patterned to achieve a helical wavefront by a vortex phase plate (VP). The 2PE and STED lasers are combined by a dichroic (D1). Fluorescence is separated from excitation and depletion light by a dichroic (D2) and collected by photomultiplier tubes (PMT). λ /2 and λ /4 are half- and quarter-waveplates used to adjust the polarization. SL, Scan lens; TL, Tube lens; SM, Scanning mirror; OL, Objective lens; CL, Condenser lens.

Experiments were performed with a custom microscope that combines 2PLSM and STED microscopy utilizing electronically synchronized Ti:Sapphire pulsed lasers (Takasaki et al., 2013). In order to achieve sufficient depletion without inducing multiphoton excitation, the depletion beam is temporally stretched to several hundred picoseconds, precluding its use for two-photon mediated photo-release of glutamate from MNI-glutamate. Therefore, in order to uncage glutamate, a third Ti:Sapphire pulsed laser was incorporated that delivered femtosecond pulses of 720 nm light (Figure 1).

### Electrophysiology, glutamate uncaging, and Ca imaging

All animal handling and procedures were performed in accordance with protocols approved by the Harvard IACUC and in accordance with federal guidelines. Acute brain slices were prepared from the hippocampus of young mice (C57BL6, ages p15–p18) as described previously (Bloodgood and Sabatini, 2007). After recovery, slices were transferred to the recording chamber in the microscope and bathed in ACSF recirculating with a small volume, closed system (10 ml). Three mM methoxy-nitroindolinyl-caged glutamate (MNI-glutamate) was added in photolysis experiments with 10 μ M D-serine to prevent glutamate receptor desensitization. All physiological experiments were performed at 34°C. Whole-cell patch pipettes were filled with a KMeSO_4_-based internal solution containing 300 μ M Alexa Fluor 594 and 300 μ M Fluo-5F, a moderate affinity calcium indicator. Cells were allowed to dye fill for 15–20 min after break-in before beginning the experiment. Photobleaching of Alexa 594 was accomplished with 2 ms pulses, whereas glutamate uncaging utilized 0.5 ms pulses. Uncaging evoked Ca transients in the spine head (ΔCa_uEPSP_) are expressed as a fraction of saturating green fluorescence obtained in the presence of 1 mM [Ca], as described previously (Bloodgood and Sabatini, 2007). This method allows comparison of Ca transient amplitudes across microscopes and laboratories since it is independent of photon collection and detection efficiency.

### Modeling of diffusive compartmentalization in dendritic spines

The morphology of dendritic spines imposes geometric constraints on the diffusion of biochemical materials within the spine and between the spine and its parent dendrite. A simple model describing transfer of freely diffusing substances between the spine head and dendrite can be obtained by approximating the spine head and dendrite as compartments of homogeneous concentration separated by a passive barrier imposed by the spine neck (Svoboda et al., 1996). The only time varying quantity in this model is the concentration in the spine head, *C*_*H*_(*t*), while the constant parameters are the cytoplasmic diffusion coefficient, *D*, the volume of the spine head, *V*_*H*_, the concentration in the dendrite, *C*_*d*_, which functions as a particle reservoir and can be set to 0, and the geometric resistance of the spine neck, *W*_*N*_, which relates the dimensions of the neck to a resistance to diffusive transfer across it. The equation governing the time course of *C*_*H*_ is then

(1) $$\frac{dC_{H}}{dt}=-\frac{D}{V_{H}W_{N}}C_{H}$$

This behavior is directly analogous to capacitive discharge in an electrical RC circuit, and there is an analogous correspondence of the geometry of a resistor and its resistance to the diffusive resistance of the spine neck. That is,

(2) $$W_{N}=\frac{l_{N}}{a_{N}}$$

where *l*_*N*_ is the neck length, and *a*_*N*_ is its cross-sectional area. Given a step change in concentration, equations (1) and (2) predict exponential relaxation with a recovery time given by

(3) $$\tau =\frac{V_{H}l_{N}}{Da_{N}}.$$

## Results

### Morphological determinants of diffusional transfer in dendritic spines

**Figure 2:**
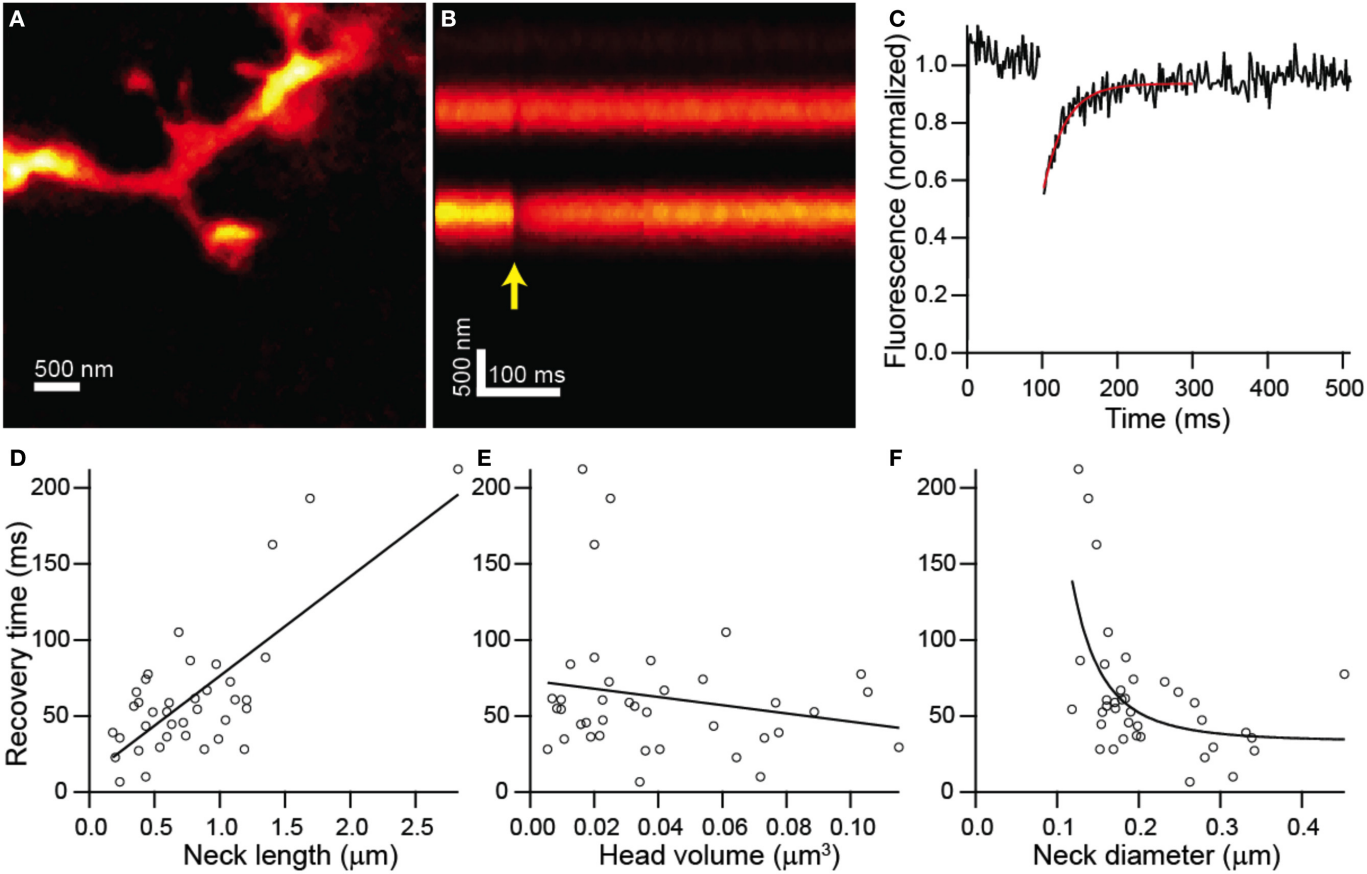
**Combined FRAP and STED-2P analysis. (A)** STED-2P image of a dendritic spine on the apical dendrite of a CA1 hippocampal neurons filled with Alexa Fluor 594 through a somatic whole-cell recording pipette. **(B)** 2PLSM linescan taken through the spine and dendrite in **(A)**. The photobleaching pulse was delivered on the spine head after a 100 ms delay (*yellow arrow*). **(C)** Fluorescence in the spine head over time quantified from **(B)**. Recovery was fit with a decaying exponential (*red line*) to obtain the recovery time constant. **(D–F)** Recovery time constants plotted against neck length **(D)**, head volume **(E)**, and neck diameter **(F)**.

In order to determine if the structure of each dendritic spine determines the time-course of passive diffusional equilibration across the spine neck, we utilized a custom-built STED-2P microscope equipped with a third pulse Ti-Sapphire laser (Figure 1; see Materials and Methods). To observe diffusional transfer across the spine neck, we performed FRAP and measured the relaxation of a step perturbation to concentration of fluorescent dye in the spine head. Spine heads were visualized under STED-2P for morphological analysis (Figure 2A), and fluorescence from the spine head was measured over time by line scanning with conventional 2PLSM (Figure 2B). During the line scan, the spine head was exposed to 2 ms illumination with laser pulses centered on 720 nm from a third mode-locked Ti:sapphire laser (Chameleon XR), producing a rapid reduction in head fluorescence attributable to photobleaching which was followed by a recovery period driven by diffusional transfer across the neck (Figure 2C).

FRAP recovery times, determined as the time constant of an exponential fit to the fluorescence, showed strong linear correlation with neck length (*r* = 0.75, *p* < 0.0001, *n* = 38) (Figure 2D), but no significant correlation with head volume (*r* = −0.19, *p* = 0.26, *n* = 38) (Figure 2E). The dependence of recovery time on neck diameter appeared non-linear and was fit by a power function, *f*(*x*) = *a* + *bx*^*n*^, with *n* = −3.28 ± 1.79 which is consistent with the value of −2 expected from modeling as a resistor (Figure 2F). The data was also fit by the same function with the exponent, *n*, fixed to −2 with a 1% increase in the residual sum of squares compared with the unconstrained fit.

To examine the validity of the simple model leading to equation (3), we compared recovery times against a parameter defined as a combination of morphological parameters, $\zeta =\frac{V_{H}l_{N}}{d_{N}^{2}}$, so that equation (3) becomes

(4) $$\tau =\frac{4}{\pi D}\zeta .$$

**Figure 3:**
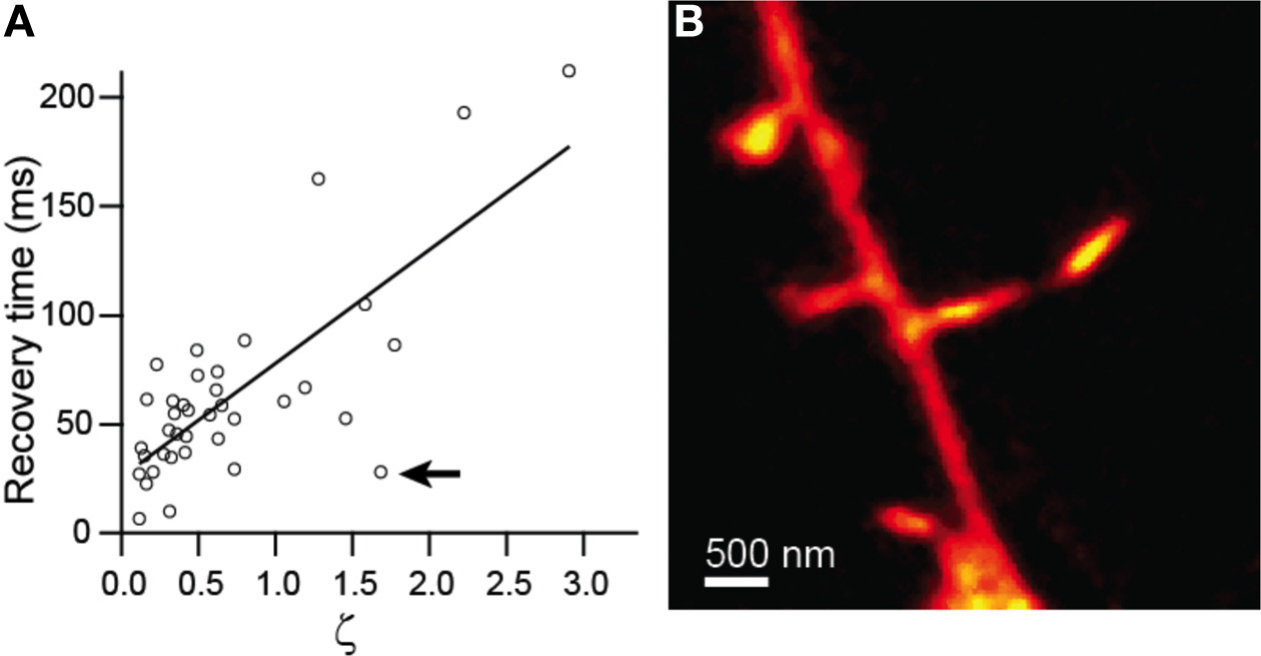
**Modeling of diffusive transfer across the spine neck. (A)** Recovery times from Figure 2 plotted against ζ. **(B)** STED-2P image of the spine producing the outlying point marked by the arrow in **(A)**.

Comparison of recovery time constant against ζ showed strong linear correlation (*r* = 0.76, *p* < 0.0001, *n* = 38) and was well fit by a line with a slope of 44 ± 7 (Figure 3A). Given equation (4), this slope value yields an estimate for the diffusion coefficient, *D* = 29 μm^2^/s. The inverse and linear correlations of Figures 2B, 3A, respectively, are consistent with accurate measurement of the morphological parameters that determine the time course of diffusional equilibration across the spine neck. Nevertheless, some dendritic spines deviate strongly from the predicted behavior, exhibiting equilibration time inconsistent with that predicted by the model. For example, the spine shown in Figure 3B and indicated in Figure 3A has a head that is similar in size and width to the neck, rendering invalid the discretized capacitor and resistor model described above. For these spines, more complex geometric models that incorporate the non-zero volume of the neck, which adds a non-zero transit time and introduces a significant reservoir of molecules, may be required.

### Impact of spine structure on synaptic potentials and Ca transients

Measurement of the subthreshold membrane potential within a dendritic spine is technically challenging due to poor electrophysiological access and the lack of optical voltage indicators with sufficient sensitivity to measure small depolarizations in individual spines. Ca influx through endogenous voltage-dependent sources, such as voltage-gated Ca channels (VGCCs) and the NMDA-type glutamate receptor (NMDAR), can be used as an indirect measurement of spine voltage. As a first step toward exploring the relationship between spine morphology and electrical compartmentalization, we combined two-photon photolysis of caged glutamate, whole-cell electrophysiology, and Ca imaging with STED-2P for morphological imaging.

**Figure 4:**
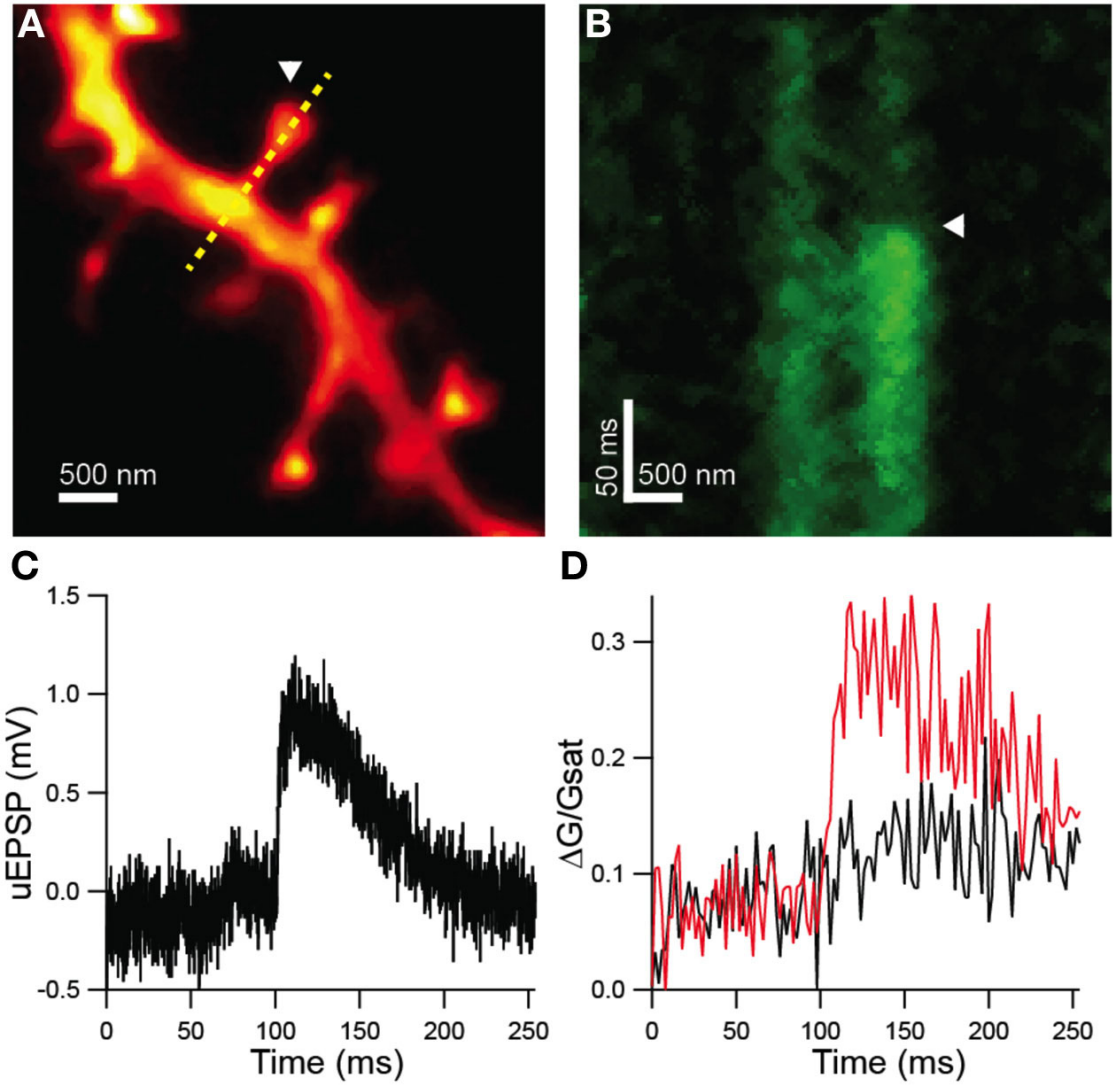
**Functional analysis of STED-2P resolved dendritic spines. (A)** STED-2P image of dendritic spines. MNI-glutamate was uncaged at a point located near a target spine (*white arrowhead*) while line scanning over the spine and dendrite (*yellow dashed line*). **(B)** Green fluorescence, indicative of Ca-bound Fluo 5F, measured in line scan. MNI-glutamate uncaging following a 100 ms delay (*white arrowhead*) produced a transient increase in green fluorescence. **(C)** Somatic membrane potential recording of the uEPSP elicited from the synapse in **(A)**. **(D)** Quantification of Fluo 5F fluorescence in the spine head (*red*) and in the dendrite (*black*) as imaged in **(B)**.

Dendritic spines were visualized by 2PLSM and STED-2P (Figure 4A) using the microscope described above. In this set of experiments, the third laser was used for the photolytic release (uncaging) of glutamate from MNI-glutamate. No shifts in holding current or increases in green fluorescence from the Ca sensitive fluorophore were observed during STED-2P imaging, likely due to the relatively low two-photon cross-section of MNI-glutamate (0.06 GM) which prevents significant uncaging by the dispersed STED pulse. Before uncaging, the amplifier was switched into current clamp mode with current injection to maintain a resting potential near −60 mV. While line scanning over the dendrite and spine, delivery of 0.5 ms illumination with laser pulses centered on 720 nm from a third mode-locked Ti:sapphire laser (Chameleon XR) generated transient increases in green fluorescence selectively in the spine head, as well as small depolarizations measured at the soma (Figures 4B–D). Green fluorescence from Ca-bound Fluo-5F was baseline subtracted and then normalized to red fluorescence from Alexa Fluor 594 (ΔG/R) to account for volume variation of the intracellular space. This metric was then normalized to the saturated G/R ratio (G/R_sat_) corresponding to the fully bound indicator as described in Bloodgood and Sabatini (2007) to obtain a measurement of Ca influx (ΔG/G_sat_) comparable across microscopes with varying collection efficiencies.

**Figure 5:**
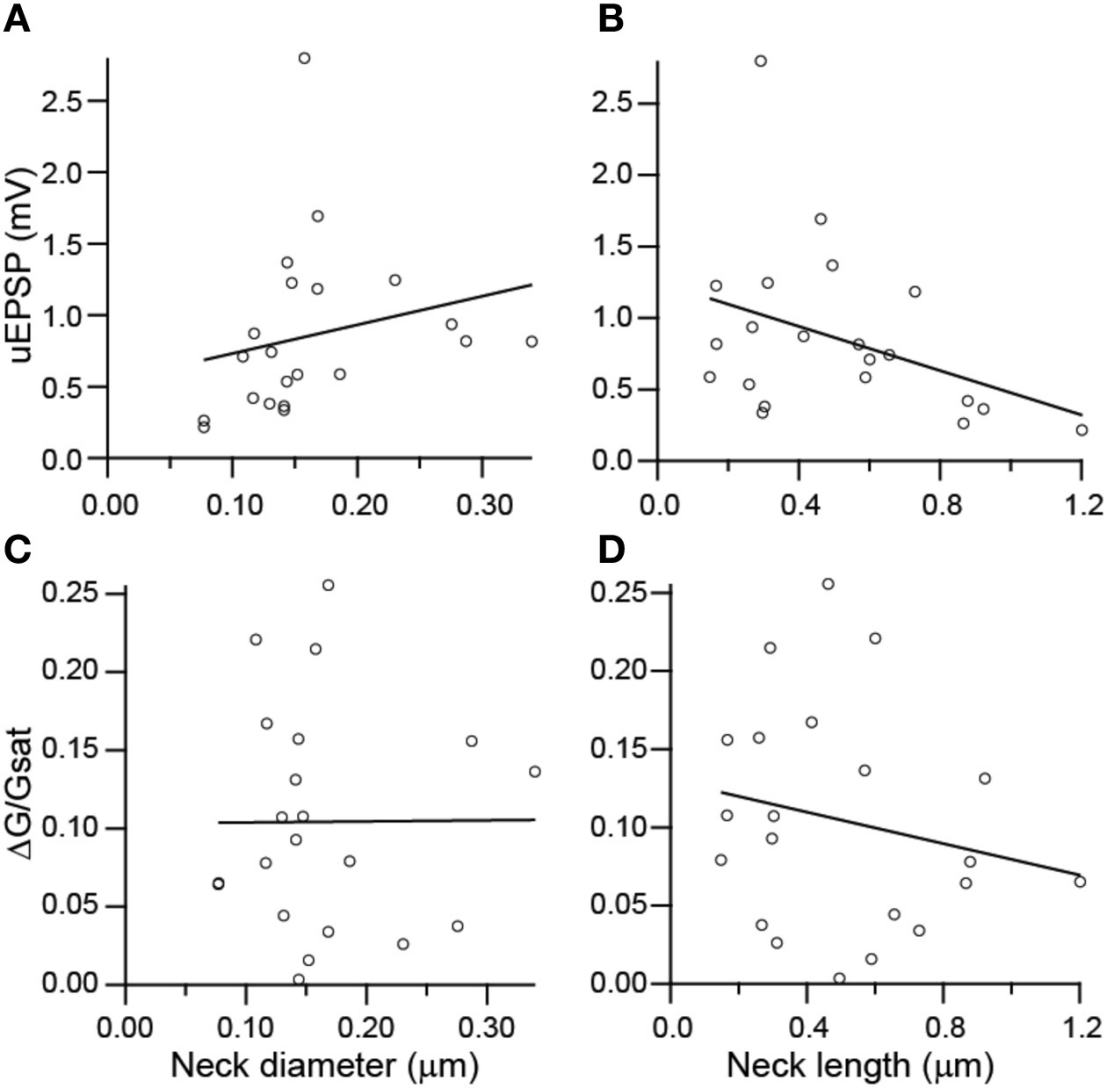
**Lack of correlations between spine dimensions, synaptic potentials, and associated Ca transients. (A,B)** uEPSP amplitude plotted against spine neck diameter **(A)** and length **(B)**. **(C,D)** Amplitude of uncaging evoked Ca transient plotted against neck diameter **(C)** and length **(D)**.

To investigate potential correlations between the amplitude of uncaging-evoked excitatory post-synaptic potentials (uEPSPs) and peak changes in green fluorescence (ΔG/G_sat_) with spine morphology, we compared these measurements against measurements of spine neck diameter and length (Figure 5). No significant correlations were observed between uEPSP amplitude and neck diameter (*r* = 0.23, *p* = 0.32, *n* = 21; Figure 5A), peak calcium and neck diameter (*r* = 0.23, *p* = 0.98, *n* = 21; Figure 5C), or peak calcium and neck length (*r* = −0.20, *p* = 0.38, *n* = 21; Figure 5D). A weak negative correlation was observed between uEPSP amplitude and neck length (*r* = −0.38, *p* = 0.09, *n* = 21; Figure 5B).

## Discussion

### Diffusive properties of dendritic spines

Experiments performed by various groups have investigated correlations between diffusive transfer of calcium, small molecules, and proteins with neck morphological parameters, and modulation of diffusive transfer in response to perturbations. Notably, activity-dependent plasticity of diffusive transfer of both small molecules and proteins has been observed (Bloodgood and Sabatini, 2005; Grunditz et al., 2008). In the Bloodgood study, synaptic activity paired with back-propagating dendritic action potentials induced changes in diffusive transfer that could not be fully explained by observed changes in spine head volume and neck length according to equation (3), thus highlighting the possible involvement of activity-induced remodeling of spine neck width. However, experiments performed with conventional fluorescence microscopy have been unable to measure sub-resolution features, such as the diameters of many spine necks, and have not been able to fully address the role of spine neck morphology in diffusive transfer. Indeed, studies have used diffusive measurements, such as the spread of synaptic Ca (Noguchi et al., 2005) or FRAP (Grunditz et al., 2008), as indirect measurements of spine neck geometry.

Our findings indicate that for a large fraction of spines, incorporation of accurate morphological measurements into a simple biophysical model predicts the passive diffusion behavior of a small molecule across the spine neck. Conversely, utilizing this model and accurate measurements of the spine neck allows for an estimate of diffusion coefficients of small fluorophores in the spine. Such analysis reveals *D* = 29 μm^2^/s for Alexa Fluor 594, a value nearly 10-fold smaller than that obtained from analysis of diffusion in Xenopus oocytes (Nitsche et al., 2004). This discrepancy may be the result of differences in methodology (FRAP vs. dye spreading) or in the local cytoplasmic environment. In support of the latter possibility, measurement of the diffusion coefficients of small molecule fluorophores in the dendrites of cerebellar stellate cells also demonstrates a near 10-fold slowing, perhaps attributable to special properties of the dendroplasm (Soler-Llavina and Sabatini, 2006).

There is a small fraction of dendritic spines for which the simple biophysical model fails to predict diffusive behavior. Recent theoretical work has clarified the approximations and limits of the biophysical model underlying equation (3) (Holcman and Schuss, 2011), and has further explored the role that subtle morphological features might play in the regulation of biochemical compartmentalization and signaling in spines. More sophisticated analysis is likely necessary to explain the diffusive properties of spines with non-canonical shapes.

### Correlation of morphology and synaptic signals

Our studies failed to find a significant correlation between the dimensions of the spine head and neck and uncaging-evoked synaptic potentials and Ca transients. Previous studies have demonstrated that many factors, including spine geometry and NMDAR subunit composition (Sobczyk and Svoboda, 2007) determine the amplitude and time course of synaptic Ca signals. In contrast, Araya et al. demonstrated that synaptic potentials were strongly inversely correlated with spine length (Araya et al., 2006) although it was not possible to determine the number of AMPA-type glutamate receptors in each spine. Our data might support the lack of a functional impact of spine shape on electrical or Ca signaling. Conversely, it may indicate the existence of counter-balancing regulatory mechanisms that adjust synaptically activated ion channels to negate the effects of spine morphology. The large variability present in the uEPSP and Ca measurements likely requires studies with very large N or more sensitive techniques to reveal significant interactions by correlative population approaches.

## Conclusion

The experiments described here demonstrate the use of STED-2P for structure-function studies in combination with experimental methods such as FRAP, whole-cell electrophysiology, caged neurotransmitter photolysis, and calcium imaging. Various theoretical and experimental findings have addressed the question of how spine morphology influences synaptic function, and the ability to probe biochemical and electrical function at an individual spine with simultaneous high-resolution measurement of morphology enables quantitative studies of such biophysical questions.

These methods could be extended to new experimental conditions or other biological systems in which nanoscale structure is of interest. For example, similar studies could be performed on dendritic spines of other cell types, such as spiny projection neurons of the striatum or Purkinje cells of the cerebellum, or on spines with perturbed structure and function in models of disease, such as tuberous sclerosis complex (Tavazoie et al., 2005) or Alzheimer's disease (Shankar et al., 2008). Alternatively, biophysical studies of dendritic spines with STED-2P could be improved upon with the application of novel experimental techniques. For example, the study of electrical compartmentalization and the role of spine geometry in regulating electrical signaling would benefit from methods able to directly measure membrane potential by an optical signal (Kralj et al., 2011; Jin et al., 2012).

### Conflict of interest statement

The authors declare that the research was conducted in the absence of any commercial or financial relationships that could be construed as a potential conflict of interest.
